# Transformation of Parkinson's care through SMART-PD (Self-Management, Remote monitoring and Timely review for Parkinson's Disease): Care pathway mapping with clinical experts

**DOI:** 10.1177/1877718X261440705

**Published:** 2026-04-17

**Authors:** Nicola Howe, Chikomborero C. Mutepfa, Sara Pretorius, Jana Suklan, Abdullahi Ali, Aisling Ponzo, Phakapan Walker, Camille Carroll

**Affiliations:** 1 NIHR Newcastle HealthTech Research Centre in Diagnostic and Technology Evaluation, Newcastle upon Tyne NE2 4HH, Newcastle upon Tyne, UK; 2 Clinical Ageing Research Unit, Campus for Ageing and Vitality, Newcastle upon Tyne, UK; 3 NIHR Newcastle Biomedical Research Centre, Biomedical Research Building, Campus for Ageing and Vitality, Newcastle upon Tyne, UK

**Keywords:** Parkinson's disease, home-based care, care pathways, remote-sensing technology, self-management, interviews, wearable technology

## Abstract

**Purpose of research:**

SMaRT-PD is a clinical decision support system (CDSS) for the home-based management and care of Parkinson's disease. It utilises remote monitoring and artificial intelligence to generate patient and clinician-facing outputs with self-management guidance, care recommendations and triggered follow-up appointments when needed. This study aimed to identify current care pathways for people with Parkinson's (PwP) and elicit healthcare professionals’ perspectives on how the introduction of SMaRT-PD would affect current care.

**Methods:**

Twelve semi-structured interviews with health care professionals working in the management and care of people with Parkinson's in NHS secondary or tertiary settings informed care pathway analysis and thematic analysis.

**Results:**

Participants described a care pathway which largely aligned with guidelines. Limitations of the existing care pathway included lengthy time to diagnosis, inconsistent care, staff shortages and challenges of appointment length and frequency. Participants outlined the potential advantages of introducing a home-based pathway including patient empowerment and education, earlier identification of patients who require an in-person review, and efficient use of clinic time. Perceived challenges to implementation of a home-based pathway were clinical and administrative. One concern was that patients and staff would miss regular face-to-face contact. A key evidence requirement for the adoption of SMaRT-PD was identified as demonstrable patient, carer and staff satisfaction.

**Conclusions:**

Participants were generally supportive of introducing a home-based pathway for people with Parkinson's, suggesting that SMaRT-PD could provide an opportunity to direct limited resources to where they are most needed.

## Introduction/background

Parkinson's disease (PD) affects over 153,000 people in the UK and is the fastest growing neurological disease globally.^1^ Standard care consists of brief (≤20 min) in-person clinic-based reviews every 6–12 months aimed primarily at symptom management, providing an opportunity for medication review and care coordination.^2^ Brief appointments and long intervals between appointments mean that symptoms are often unreported or overlooked as their declaration is dependent on patient understanding and recall.^3,4^ Opportunities for patient or carer education on recognising and managing complications are limited^3,4^ and access to specialist expertise is variable, constrained by geography and resources.^5^ The costs associated with Parkinson's disease, both to the healthcare system (e.g., admissions, medication) and to the patients themselves (e.g., travel to appointments, ability to work) are higher than for those without the condition and tend to increase as disease advances.^6,7^

Congruent with NHS England priorities such as hospital at home models^8,9^ and patient initiated follow-up (PIFU),^10,11^ home-based care with remote monitoring offers an opportunity to reduce unplanned hospital admissions, increase bed capacity and provide patients with quality, individualised care in the comfort of their own home.^12–16^

SMaRT-PD (Supported self-Management, Remote monitoring and Timely review for Parkinson's Disease) is a clinical decision support system (CDSS) designed for the management of Parkinson's disease. It incorporates rules-based decision trees and integrates multimodal data including patient and carer-reported outcomes, wearable sensor data from the wrist worn Parkinson's KinetiGraph (PKG^TM^),^17^ and routine care records to generate tailored outputs for patients and clinicians. These outputs include a status report, personalised self-management guidance, and care recommendations including indications of when healthcare contact is needed. Integration of SMaRT-PD pathway within a home-based care pathway facilitates supported self-management, timely triggered review and objective remote monitoring.

Co-developed with people with Parkinson's (PwP) and their care partners,^2^ SMaRT-PD is currently being piloted as a service improvement with approximately 280 eligible patients in Plymouth, West Devon, and East Cornwall.^18,19^ The main goals of SMaRT-PD are for patients to improve their understanding of Parkinson's disease and their ability to self-manage whilst empowering them to request clinical support when needed. The main goals for healthcare organisations are to provide targeted and timely clinical input and prevent deterioration, avoidable admissions, institutionalised care and complications, while improving staff wellbeing and reducing social care needs.

Here we present findings from work conducted by methodologists at the NIHR HealthTech Research Centre for Diagnostics and Technology Evaluation (NIHR HRC DTE). The study objectives were to explore current care pathways for PwP and capture healthcare professionals’ (HCPs) perspectives on the practical implementation of SMaRT-PD, including the perceived effects on the health system, healthcare providers and patients, potential barriers and facilitators to the implementation of SMaRT-PD and evidence requirements to support successful integration.

## Methods

This study followed a protocol (unpublished) and is reported with reference to COREQ, (Consolidated Criteria for Reporting Qualitative Research).^20^ COREQ is a 32-item checklist specifically designed to improve the completeness and transparency of reporting in qualitative studies that utilise interviews and focus groups.

### Care pathway analysis

To identify the current care pathway for PwP and given the heterogeneity of service delivery, a high-level care pathway analysis (CPA) encapsulating commonalities between services was conducted following the methods adopted and developed by HRC.^21^ A process map was developed using Lucid Chart.^22^

### Semi-structured qualitative interviews

We performed semi-structured interviews with HCPs who have responsibility for managing or delivering care to PwP, to validate the proposed care pathway, and address the study objectives.

### Participant selection and recruitment

We intended to perform up to 10 interviews with clinical experts who oversee the management and care of PwP within NHS secondary or tertiary care in England. This included professionals from geriatrics and neurology, such as nurses, registrars and consultants with varying levels of experience. However, due to service variability we expanded this to include up to 15 clinical experts. We recruited from a diverse mix of rural and urban settings in England, spanning university and teaching hospitals, district general hospitals (DGH) and from specialist neuroscience centres.

Between July and October 2024 potential participants were identified through contacts of the study team, primarily those known to author CC, and sent email invitations and participant information sheets. Snowball sampling methods^23^ were employed thereafter. The study was also advertised via the CHAIN network,^24^ a support network for people working in health and social care, and through the Parkinson's Excellence Network newsletter. Participants contacted the authors if they wished to take part and completed a consent form (n = 11) or gave verbal witnessed consent (n = 1) prior to the interview taking place. Thirteen email invitations were sent from which seven participants agreed to take part. Three Participants were suggested by colleagues who took part, and two Participants contacted the researchers after seeing the CHAIN and Parkinson's excellent network newsletter. Participants were not compensated for participation, and none withdrew from the study.

### Ethical approval and consent

The study received ethical approval from the Newcastle University ethics committee on 17^th^ June 2024 (Ref: 48295/2023). An amendment to this approval was granted on 6^th^ November 2024 to increase the number of participants from ‘up to ten’ to ‘up to 15’.

### Patient and Public Involvement & Engagement (PPIE)

The study protocol, participant information sheet and consent form were reviewed by the NIHR HRC Insight Panel.^25^ The insight panel was formed to ensure public accountability and transparency regarding the work of NIHR HRC DTE and provide an opportunity for the public to influence change in the healthcare issues which concern people most. Documentation was amended as applicable following feedback. The HRC Insight Panel also provided comments on the overall study design which were shared with the SMaRT-PD study team.

### Data collection

Semi-structured interviews followed a topic guide (supplementary file) collaboratively developed by all authors and informed by relevant literature and guidance on the care of PwP. The topic guide covered interviewee characteristics, the current Parkinson's care pathway, and participants’ perspectives on the SMaRT-PD pathway/CDSS technology (including clinical need, potential barriers and facilitators to adoption and evidence requirements). A standardised description of the SMaRT-PD pathway was read to each participant. The topic guide was piloted during an interview with author CC following which no further changes were made.

Interviews were conducted online between July 2024 and January 2025, led by author NH with at least one other author (PW, SP, CCM, JS or AA) present. Interviews lasted between 40 and 60 min and were audio-recorded with the consent of participants. Detailed notes were taken during interviews using a Rapid Assessment Process Sheet (RAP) as described in rapid qualitative research processes.^26,27^

### Qualitative data analysis

Automated transcripts were reviewed and corrected by the authors to ensure accuracy before being imported into qualitative data analysis software NVivo version 14.^28^ Four authors (NH, PW, SP & AA), following a realist epistemological position independently and inductively^29^ coded two transcripts using thematic analysis methods^30^ to pilot the coding approach. Codes were then discussed and agreed by three authors (NH, PW & SP) before being consolidated in a codebook^31^ in NVivo. All subsequent coding was completed by author NH using a ‘structural’ or ‘theoretical’ approach^29^ ^p^.137^30^ guided by the codebook with amendments and new codes added as necessary. Codes were then grouped into themes in NVivo following Braun and Clarke's phase 3 methodology and reviewed by authors NH, PW and SP in phase 4.^30^ The RAP supported reflexive analysis throughout. Participants were not involved in cleaning or analysis. The identified themes and data from NVivo underpin the narrative of this manuscript.

## Results/findings

### Care pathway analysis

**Figure 1.**
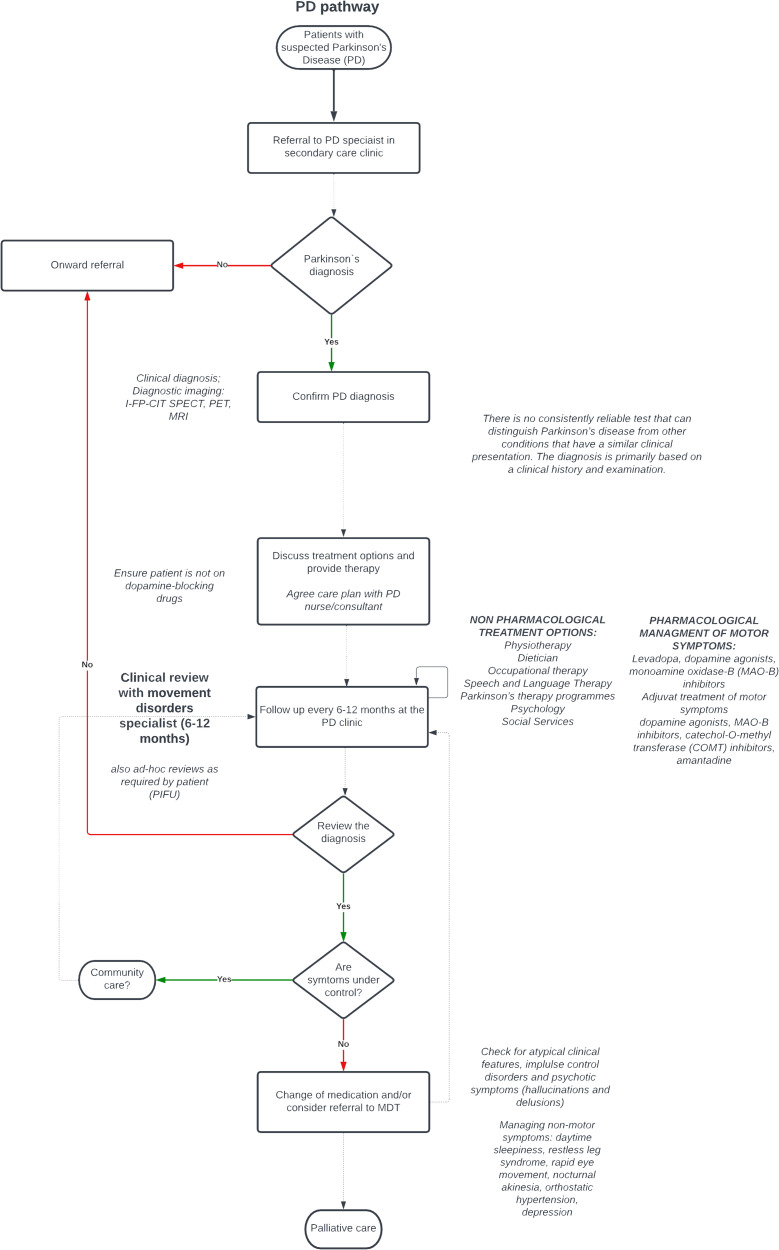
Parkinsons pathway.

A high-level care pathway^21^ was developed from a review of NICE guidelines for the care of PwP^32–34^ supplemented by guidelines from SIGN,^35^ Parkinson's UK,^36^ The Royal college of occupational therapists,^37^ the association of UK dietitians in association with Parkinson's UK^38^ and UK Parkinson's excellence network.^39^ Data from guidelines were extracted into a summary table and a care pathway was drafted and refined following the interviews. All participants reported adherence to guidelines although most did not routinely ascertain the stage of disease at the time of diagnosis. The final validated care pathway is available in Figure 1.

## Semi-structured qualitative interviews

### Interviewee characteristics

We conducted twelve interviews with HCPs responsible for the care or management of PwP within the NHS. The participants interviewed were from either a neurology or geriatric background, had a median Parkinson's care experience of 14 years (range 4–30 years) and covered 10 NHS trusts. Ten of the participants qualified for clinical practice in the UK. List sizes ranged between 200–2000 patients. Interviewee characteristics are displayed in Table 1 and Table 2.

**Table 1.** Interviewee job roles and duration of experience in Parkinson's care.

Role	Number of participants	Years experience in Parkinson's disease
Geriatric medicine registrar	2	4–7
Consultant neurologist	3	10–18
Consultant geriatrician	3	11→20
Neurology specialist nurse	1	8
Parkinson's advanced nurse practitioner	1	>16
Parkinson's specialist nurse	1	9
Parkinson's disease nurse consultant	1	>30
TOTAL	12	Range 4- 30

**Table 2.** Included site characteristics.

Site location	Centre type	Centre consultant specialism	Centre nurse specialism
Bath	District General Hospital	Geriatric medicine & neurology	Parkinson's or neurology depending on location
Brighton	Regional Neuroscience Centre (RNC)	Neurology and Parkinson's specialist	Parkinson's specialist
Coventry and Warwickshire	University Hospital & cover for general hospitals	Neurology	Parkinson's specialist
Dorset	District General Hospital	Geriatric medicine	Parkinson's specialist
Kings Lynn	District General hospital	Neurology	Neurology
Kings Lynn/Norwich/Cambridge	University Hospital	Neurology (& Geriatric medicine registrar)	Parkinson's or neurology depending on location
Liverpool	Regional Neuroscience Centre (RNC)	Neurology	Parkinson's specialist
London	Regional Neuroscience Centre (RNC)- Specialist Parkinson's centre	Neurology	Parkinson's specialist
North Cumbria	District General Hospital- specialist Parkinson's service (encompassed by elderly care)	Geriatricians specialising in Parkinson's (some formerly neurology)	Parkinson's specialist
Northumberland	District General Hospital (plus clinics across Northumberland/ Cumbria)	Geriatricians	unclear
North Wales	District General Hospital	Geriatricians	Parkinson's specialist

## Current approach and pathways for managing PwP

### Service set up

While participants reported that their care practice for PwP largely followed NICE guidelines, some variation in clinic set up was described, seemingly unrelated to centre type. Care was overseen by either neurology or geriatric teams, some of whom had specialist roles or interest in Parkinson's (Table 2). Day-to-day patient care was either overseen by nursing teams supported by consultants, or consultant-led with nurse support. Referrals depended on the hospital or trust managing cases, patient preference, and age, with younger patients more often being referred to neurology.

In some areas patients were ‘discharged’ to community care post diagnosis, whilst in others community care was supplementary to clinic appointments.

### Diagnosis and follow up

Patients were typically referred by their GP to either neurology or geriatric care. Initial diagnosis by a consultant usually occurred after several brief appointments lasting 30–40 min each. Nurses might follow up with a slightly longer, more informal ‘newly diagnosed workshop’ where patients ask questions or receive signposting and information packs.

Follow up appointments were generally scheduled every 6 months, often occurring every 8 to 9 months due to delays. Some services had one-stop-multi-disciplinary clinics, while others alternated nurse and consultant appointments reducing time between follow up appointments. Follow up appointments were described as too short (30–45 min for nurse-led clinics and 15–30 min for consultant-led clinics) and “chronically overrunning”.

Follow up appointments generally included blood pressure measurements, scoring of non-motor symptoms, discussion of medication side effects and review of motor and intrusive symptoms. Some participants used pro-formas or checklists, such as those provided by Parkinson's UK, whilst others thought these too performative. All participants were focussed on what was currently most important to the patient. Some discussed how patients could best self-manage their care.

Patient initiated follow up (PIFU), reactive or flexible appointments and the ability to contact staff between appointments were mentioned by most participants. Patients who were the most persistent or knowledgeable about how to ‘wield the system’ tended to access and benefit from PIFU more than others.

### Aspects of the current pathway that work well

The relationship between healthcare professionals and patients was described as friendly; built on familiarity, with both patients and HCPs getting a lot out of face-to-face contact. Some participants described appointments as a highlight of their work.

Participants cited holistic and responsive care as a key strength of the current Parkinson's care pathway. For some this meant addressing all of a patient's needs through multidisciplinary clinics. For others it meant being responsive to individuals’ needs relative to their social circumstances, carer needs and home care situations, providing support in the community where available, and reminding patients about things such as driving and exercising. Responsiveness was characterised by accessibility (compared with other health services) and availability of the care team between appointments, ensuring that symptoms did not go unchecked for too long. Participants also emphasised the importance of providing reassurance and education, giving patients both understanding and acceptance of their diagnosis.

### Challenges in the current pathway

Although participants felt they provide a good service in challenging circumstances, they described several difficulties in the management and care of PwP including the anxiety-inducing delay to diagnosis, followed by what was often described as ‘patchy’ care. Access to services, particularly in the community was largely dependent upon geography and the NHS trust(s) involved. Communication and co-ordination of care between trusts or services, particularly between regional neuroscience centres and the community was highlighted as problematic. In the most concerning cases, poor communication was said to lead to ‘patients falling through the cracks’.

Understaffed clinics struggle to maintain 6 monthly follow up intervals and have limited capacity to respond to patient calls and emails between appointments. Follow-up appointments (particularly in consultant-led clinics) were described as too short to cover everything that a patient might wish to discuss or to provide thorough oversight of all potential symptoms. Referrals to over-stretched services also led to delays. Waiting times for diagnosis and follow up appointments and lack of time to consider or implement new treatments or research were attributed by participants to staff shortages brought about by retirement, increasing patient populations, lack of funding for allied health professionals, long-term sickness and difficulty in recruiting trainee HCPs to more rural posts.

### SMaRT-PD pathway

**Figure 2.**
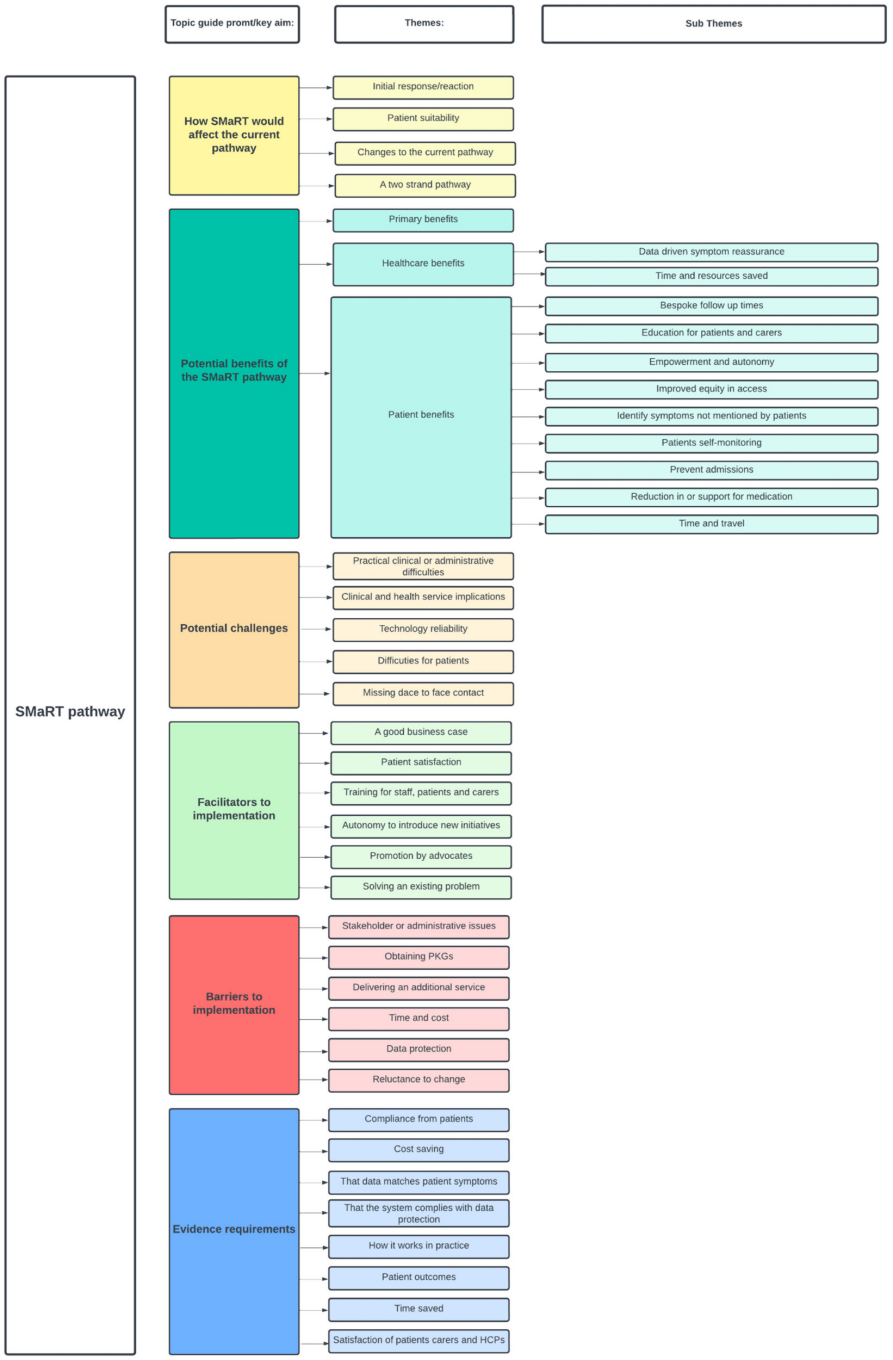
Smart theme diagram.

Themes (and sub themes) related to the SMaRT-PD pathway, identified during analyses are presented below in a narrative and presented visually in Figure 2.

When presented with a description of SMaRT-PD most participants were positive about its potential to monitor patient symptoms, enable more bespoke follow ups and provide education to patients. Key themes included patient suitability, changes that would need to be made to the current pathway, and the potential for a 2-strand pathway.

### Patient suitability

Most participants instinctively reflected on which patient groups the SMaRT-PD pathway would be appropriate for, and which it might not suit. Initially, participants thought that SMaRT-PD would be less effective for elderly patients in later stages of Parkinson's compared with younger, less symptomatic patients who are more confident using technology. However, upon reflection, most participants acknowledged that the picture is more nuanced; younger patients at the diagnosis stage may experience significant anxiety and benefit from face-to-face contact. People who live rurally and who are older might benefit from fewer trips to the clinic, particularly as the level of comfort with technology increases over time as older populations continue to grow. The type of patient who would be suitable for SMaRT-PD depends on factors including individual circumstances, personality, diagnosis stage, symptoms, cognition, age, location, grasp of technology, medication regimen, and patient preference

*“It would take a little bit of teasing out, I think, to work out who those people would be, and it might not necessarily be obvious, you know, for example, at the time of diagnosis. I think there's a group of patients who would find it quite frightening and would need a lot of reassurance about it to start with”.* (P01 Geriatric medicine registrar working only in Parkinson's)

### Changes to the current pathway

Participants identified that SMaRT-PD may bring about a reduction in face-to face clinic appointments, allowing HCPs to respond when clinically necessary rather than at fixed intervals. For services already offering good access to the Parkinson's team or following a PIFU model, participants thought that the pathway would not change significantly. Phone or video consultations introduced during COVID-19, particularly for some ‘younger’ patients, were seen as likely to continue.

Participants highlighted that symptoms in newly diagnosed patients may progress further than anticipated with longer gaps between visits, necessitating careful management of medication and side effects. Time would be required to review data from attending patients, and additional appointment slots would need to be reserved for patients whose data indicated that they required a review. Uncertainty remained around the management of incidental findings, such as depression and which service would take ownership of co-existing conditions:

*“If we do pick up someone with depression on the HADS [Hospital Anxiety and Depression Scale], what are you going to do? Is that just going to be a letter back to the GP, by the way, your patient has scored X on the HADS scale. Could you please look into this? Or are we then going to follow that up ourselves and say, actually, can you come as an extra to the clinic? Because we need to talk to you about your depression that you've reported in your scale.”* (P05 Consultant geriatrician)

Reduced routine contact could limit opportunities to build strong relationships and truly understand patients’ conditions. Participants suggested that an interesting unintended consequence of reduced clinic appointments could be that all face-to-face meetings would be with patients when they are experiencing problems, potentially making clinics less personal and more transactional.

*“There are advantages to see people routinely. So, you get to know them, get to know their families, you get to get to get a feeling of the trajectory of their illnesses, and it develops of a relationship, whereas if you're just seeing them when there are crises, it somehow changes the sort of the dynamic of the relationship, to some extent”*. (P11 Consultant geriatrician)

### A two-strand pathway?

Although participants had identified that there were some patients for whom the SMaRT-PD pathway would not be suitable, most felt confident that they could divide their clinics into two strands so that the SMaRT-PD pathway could be targeted at individuals most likely to benefit. Implementing such a model would require changes to clerical and administrative practices. For example, routine clinics could be cancelled in favour of patients requiring follow up, rooms would need to be booked or re-allocated. Despite administrative concerns, a two-strand pathway could drive improvements, efficiencies and cost savings in an ever-stretched service.

*“There are people I could absolutely imagine would buy into this…people who are kind of highly motivated and activated, want to take on responsibility, and it would be super for them. So yeah, in some services, almost everyone in others, maybe a subset. And then there's obviously logistics around that, but not insurmountable. I don't think.”* (P07 Geriatric medicine registrar)

### Benefits of the SMaRT-PD pathway

Participants speculated on the benefits that the SMaRT-PD pathway could bring to patients and their management and care.

### Healthcare benefit- data driven symptom reassurance

Participants identified a primary benefit of SMaRT-PD as the ability to use objective, patient-level data from remote monitoring to enhance clinical decision-making for people with Parkinson's disease (PwP). They described the benefits of being able to compare reported symptoms with collected data to check for correlation.

*“You know, what I come across is when I call them, and sometimes they think that they're getting worse. But if you actually see them in the clinic, they're not that bad. So that we get information, and then at least we could reassure them that's not the case, you know. Or maybe we could adjust the medication”.* (P04 Neurology specialist nurse)

Systematic data collection could also establish individual baselines allowing for the tracking of disease progression over time and perhaps help improve the understanding of ‘off times’ pattern and dyskinesia to inform individually tailored care. Systematic gathering of data could also be valuable for research and clinical audits, with a database that can be retrospectively referred to, both on an individual and population basis.

### Healthcare benefit- time and resources saved

Reducing the number of routine follow ups was identified as a potential source of time and cost savings, both in clinics and community settings. Administrative and support time could also be saved, with fewer letters sent and appointments made, and fewer rooms booked. Stretched resources could be redirected to those that really need them, and patient's appointments could be structured around data rather than pre-determined questions which may not be applicable. HCP time could be better used elsewhere, for example developing initiatives that would benefit PwP.

*“It is going to reduce the work time, and admin support time, because if they’re not seen every six months, you know, even secretaries don't need to type the letters or send them to the GPs. And cost as well, because every clinic, has a cost attached to it.”* (P04 Neurology specialist nurse)

### Benefits to patients

Participants identified numerous benefits to patients including reduction in travel and equity of access to healthcare. However, the primary benefits were considered to be patient empowerment, enhanced patient autonomy and increased education and understanding around Parkinson's. Using data to demonstrate baseline, on and off periods and features of dyskinesia and dystonia can help patients to understand their condition earlier and identify complications before they occur. The more a patient understands their condition, the greater their ability to self-monitor and manage at home. Visualisation of data is already popular with patients and carers, allowing patients to gather their thoughts pre-appointment, discuss their own data and engage in decisions on treatment during appointments.

*“What I found quite fascinating is that the people that are having this PKG recording often really want to see the result in the clinic, and they want to see the graph.”* (P08 Parkinson's disease nurse consultant)

Education and self-management through understanding of one's own condition was strongly linked to the concept of empowerment and autonomy.

*“We certainly have lots of patients who want who manage their disease by just really understanding it. And actually, it gives them a kind of framework, as opposed to really understand what's happening, how their medication works, how their body is responding. And I think for some people, that would be really beneficial, definitely, to give them that autonomy.”* (P01 Geriatric medicine registrar working only in Parkinson's)

Self- management could have healthcare benefits for patients by uncovering symptoms that are not visible, that patients are unable to identify themselves or that patients are less likely to disclose. Identification of previously unreported symptoms could provide a safety net, with treatment increasing quality of life.

“*There might be things that you uncover there that you wouldn't have otherwise uncovered because the patient doesn't volunteer that in the clinic, and if you don't ask about it, you won't necessarily hear about it…* *you're going to avoid people slipping through the net who might be severely depressed but you haven't asked about this, or they haven't told you about it. I think the non-motor symptom scale, again, it might help flag up problems.”* (P05 Consultant geriatrician)

Early symptom or deterioration identification was perceived as a way to prevent hospital admissions by enabling timely intervention or medication adjustments.

“*We can reduce admission to hospital if we get direct updates of their symptoms. So then at least we could advise or adjust the medication accordingly, so they don't come to hospital regularly.”* (P04 Neurology specialist nurse)

It might also support more precise medication adjustment based on data collected, helping to reduce side effects associated with medicines optimisation.

“*My gut feeling is that people would potentially end up with less levodopa medication overall if we can demonstrate that somebody is doing very well, we’ll be less inclined to increase their dose when they come to clinic. If we can explain that to them using robust data. And I think not having as high dose of levodopa early on, or particularly as they're getting frailer, feels like a good thing…people are going to get less in the way of side effects and cognitive side effects.”* (P01 Geriatric medicine registrar working only in Parkinson's)

From a practical point of view, self-management at home could reduce unnecessary travel and clinic visits, easing the burden on patients, particularly for those in remote areas, working full-time or who perceive routine visits as unnecessary.

“*I guess it feels like it would reduce sort of unnecessary burden on people coming up to hospital appointments that they don't want or need.”* (P07 Geriatric medicine registrar)

Replacing standard follow-up appointments with evidence informed, patient-triggered reviews could also reduce health inequalities, particularly in rural or underserved areas.

“*What is quite important, I think, is to be sure that we equally access all the population. We can communicate and make available all we have to all patient populations, because it's unbelievable, the inequalities…I have a patient in a small village. Sometimes they struggle to travel here, so I need to speak to them on the phone”.* (P10 Consultant Neurologist)

### Potential challenges

Participants addressed the potential challenges associated with the introduction of the SMaRT-PD pathway, categorised into the following themes: practical clinical or administrative difficulties, clinical implications, difficulties for patients, missing face to face contact and technology reliability.

### Practical clinical or administrative difficulties

Some participants wondered who would be responsible for administrative tasks associated with the roll out and management of a home-based pathway. They questioned how community teams would be included, and whether they would have access to PKG^TM^ data.

“*What it could do is, is end up funnelling more questions to me…if only I've got access to the wearable device and they've gone to see the patient and the patient says they're having problems, it may just end up with a letter to me saying, what does the last PKG show? Could you give us some advice? It's just unforeseen knock-on effects I could envisage, for example.”* (P03 Consultant neurologist)

The requirement for time to review the PKG^TM^ data was mentioned by most participants. While fewer routine follow-up appointments could save time, participants thought that this would be offset by the time taken to review data and provide feedback although they might benefit in the long term from the data collected. There were questions about who would monitor and analyse the data and how long this would take for each patient.

“*I need then the time to go through the results of the questionnaire, sending a letter to the GP and the patients with the disadvantage of not seeing the patients in person…So this is extra time that we need to squeeze in somewhere…dictating a letter, going through the letter, approve the letter.”* (P10 Consultant Neurologist)

Some participants noted the potential administrative difficulty of overhauling clinic bookings and changing the pathway, with previous experience telling them that this could be a problematic or manual task requiring training for clinic secretaries or booking teams working across multiple trusts.

“*The challenge is trying to integrate it into the bookings pathway, making sure that the bookings team understood the pathway, and they understood how the clinics would be booked and initiated, and then that would have to cross over different healthcare trusts.”* (P09 Consultant Neurologist)

Finally, participants thought that remote monitoring could raise patient expectations for treatment of symptoms or fluctuations identified by PKG^TM^ monitoring and that time would need to be found to discuss observations or answer questions. Once something was identified, HCPs would be ‘obliged’ to do something about it.

### Clinical and health service implications

Participants identified potential challenges for clinical practice such as compliance with NICE or Parkinson's UK guidelines, current processes and procedures and whether they would miss symptoms by not seeing patients face-to-face. In-person consultations were seen as essential for training Parkinson's nurses, providing opportunities to observe motor and non-motor symptoms directly and to learn from consultants. Extended gaps without in-person assessments could limit learning opportunities and reduce the ability to detect nuanced fluctuations in the condition. Some symptoms, such as swallowing difficulties or bowel issues were viewed as beyond the scope of remote monitoring.

There was concern that important context around observations would be missing without face-to-face consultations

“*It's not the quality of the data, it's also how the patients express the problem.”* (P10 Consultant Neurologist).

Participants referred to ‘probing’ and ‘delving’ to identify the circumstances around fluctuations or symptoms, for example, some symptoms exhibited in the data would need to be ‘correlated’ to patient activities to avoid misinterpretation e.g., exercise being mistaken for dyskinesia.

“*Sometimes a whole clinic appointment can be around postural hypotension, particularly in people who are a bit more complicated. You know what's caused that fall? Was it a fall? Do you remember it? Do you recollect it? And you know what precipitated it? Where were you at the time? Were you standing? Were you sitting? And we don't really have a specific from the questionnaires, that capture these sort of questions in relation to something like that. That can make a big difference with treatment decision making.”* (P06 Parkinson's advanced nurse practitioner)

Measurements such as blood pressure and weight would still need to be captured in clinic. Furthermore, participants referred to cognitive, non-physical or non-motor symptoms that could be missed, for example loneliness, apathy or signs of cognitive decline as they are generally revealed through in-person discussion with carers or partners.

“*I think there's loads of non-motor symptoms that are very important, and they may well be being covered. But, we talked earlier about constipation, and swallowing, cognition, hallucinations, all this sort of neuropsychiatric stuff, sleep, you know, pain, I think a lot of these things, if we don't ask, we don't know about it. I think bladder symptoms as well are often quite under recognized.”* (P07 Geriatric medicine registrar)

One interviewee questioned whether 6 days’ worth of data was enough to establish a representative baseline measurement for a condition that fluctuates.

### Difficulties for patients

Perceived difficulties for patients revolved around the anxiety that PwP experience, and the potential for patients to ‘fall through the cracks’.

Thresholds for follow up triggers would need to be robust to ensure that patients who were most stoic, least likely to contact their care team, most apathetic, cognitively impaired, socially isolated or with psychological problems were not unintentionally disadvantaged. There was concern that interventions, effective only for patients already adept at managing their health, could inadvertently widen health inequalities.

“*I worry sometimes that if an intervention works for the people who are already best at managing their health, I guess you can widen that inequality. Or kind of improve outcomes quite a lot for some people, and then actually outcomes could worsen for others.”* (P07 Geriatric medicine registrar)

Increased monitoring or completion of symptom scales had the potential to exacerbate anxiety in already anxious patients by drawing attention to their symptoms.

“*I'm not sure as well, whether that kind of thing would raise anxiety with patients having it done so frequently, have my symptoms deteriorated? Am I going downhill? So you know, thinking about their well-being and how they're feeling in themselves, if they're permanently thinking about scores and monitoring of their symptoms. Does that make them feel defined by Parkinson's rather than living with it?”* (P06 Parkinson's advanced nurse practitioner)

### Missing face to face contact

Participants emphasised the value of face-to-face contact which allows the development of a valuable relationship between HCP and patients and carers. Many therefore expressed a reluctance to reduce in-person interactions, for clinical or practical reasons but also because they enjoy clinic visits. Several participants mentioned a preference for ‘chatting’ to patients over reviewing data.

“*I can get a feeling for how my patients are getting on reasonably well with a 10 min chat… and to try and do that remotely, without seeing the patient, I find that rather, rather dispiriting. Quite honestly, I love my time in the clinic, with patients. It's sort of what I go to work for. And the sort of dry Excel spreadsheets looking at PKGs I find much less interesting.”* (P03 Consultant neurologist)

Some participants spoke from the perspective of patients, describing how much patients value their clinic visits and interactions with their Parkinson's team.

“*They need a contact, they need a conversation. They need that coming to hospital. Well, you know, for them, it's the highlight of the day. They don't go out that much. A lot of people that are anxious still get something out of talking to their doctor or nurse face to face, and then just being able to say one human to another, don't worry. You're doing really well. Please, don't worry about this. It's normal or whatever, and that's something that you would lose in a questionnaire type situation”* (P08 Parkinson's disease nurse consultant)

Seeing carers in face-to-face appointments was thought to provide triangulation of data received from patients and clinical observations. Some participants questioned what input carers would have in the remote monitoring system, and whether carers had input into the design of SMaRT-PD.

“*Sometimes you're getting one answer from the person with Parkinson's as to how things are. And then you sense sometimes from body language that the carer has a different view, and that obviously can be a bit challenging to sort of sensitively unpick. It may be that has been captured, but if I had any immediate concern, it would be, are they getting a voice in this? Just sometimes a loved one has a slightly wider perspective on how things are changing.”* (P07 Geriatric medicine registrar)

### Technology reliability

Minor concerns were expressed regarding the use of PKG^TM^ and the completion of questionnaires. These were based on prior experience or expectations of how patients could manage or access technology depending on their age, familiarity with technology or rural location.

“*They say that they can manage it, and they can use the app on their smartphone, but when it comes to it, they can't, or they don't know what a smartphone is, or their internet is terrible, or they actually don't have an internet connection. We need people to remember them and wear them and not lose them, and the technology all to link up and work together. We've had a tricky experience of that recently.”* (P01 Geriatric medicine registrar working only in Parkinson's)

### Adoption of the SMaRT-pd pathway

Interviews addressed perceived barriers and facilitators to adoption of the SMaRT-PD pathway as well as the types of evidence that would encourage practitioners or services to implement a home-based pathway. Key themes are summarised below under the key headings of facilitators, barriers and evidence requirements, understanding the artificial intelligence and settings and logistics of SMaRT-PD piloting.

### Facilitators to implementation

Some participants had prior experience of introducing a new technology such as PKG^TM^ into care pathways. Key facilitators to implementation included demonstrable patient satisfaction, training for staff, patients and carers, having the autonomy to introduce new initiatives, promotion and advocacy (for example by Parkinson's UK) and alignment with existing service gaps. The most frequently mentioned facilitator was presentation of a business case to relevant stakeholders with health economic data.

Potential cost and time savings through reduced outpatient appointments were seen as the most attractive proposition for managers or commissioners. One interviewee considered that creating additional outpatient clinics specifically for the SMaRT-PD pathway might create a good business case for recruiting additional staff, as well as administrative or secretarial support, improving the Parkinson's service overall.

Ultimately, SMaRT-PD would have to demonstrate cost neutrality or savings to be considered for implementation. Only one interviewee (at a regional Parkinson's centre) indicated that they had a strongly positive relationship with managers or commissioners and all participants believed that improvements in care quality or patient satisfaction were perceived as secondary to the financial implications by commissioners or managers.

### Barriers to implementation

Resistance to change, especially among staff and patients with limited digital skills or access to technology, was identified as a key barrier to adoption. Other stakeholders such as hospital or trust management may be unreceptive to change or hold conflicting views on how to implement new technologies or services. This combined with the need for staff training, bureaucratic and governance requirements and inequities in accessibility or resources across different communities or Trusts can hinder the adoption of new technologies. Some participants thought that obtaining the PKG^TM^ would be too difficult; that they are too costly, and additional payments for extracting data or having to ‘buy in’ to a contract was off-putting. Participants also questioned whether SMaRT-PD constituted an additional or replacement service raising concerns about staff capacity for service development and potential additional appointments generated as symptoms are identified. Time (and associated cost) to read reports, review data and the obligation (even remotely) to deliver follow up care based on these were also identified as key considerations. Some participants highlighted data protection requirements in terms of storage and compliance from NHS infrastructure as a potential barrier to adoption.

### Evidence requirements

By far the most important evidence requirements were demonstrable satisfaction and compliance from patients, carers and healthcare professionals. Participants felt that receiving qualitative data on patient satisfaction or speaking to health professionals who already implement the SMaRT-PD pathway would be the best way for them to gauge whether their service was suitable for adaptation to a SMaRT-PD pathway.

“*If it had been piloted elsewhere…* *perhaps chatting to the clinicians who've actually used it, and the sort of service they've got to see if it's comparable”* (P12 Consultant geriatrician)

Participants highlighted the importance of engaging a wider group of stakeholders before implementing the SMaRT-PD pathway. These included secretaries, the clinic booking office, the IT service, governance and financial teams and GPs.

Other evidence requirements were demonstrable time and cost savings, evidenced through published literature, alignment between CDSS data and patient-reported symptoms, and a positive effect on patient outcomes (e.g., identification of exacerbation, prevention of admission).

### Understanding the artificial intelligence

Although participants expressed general trust in the decisions made by the CDSS, they felt that they would like at least some understanding of how the artificial intelligence driving the system works, not least because this would allow them to promote and explain it to patients.

“*I would like to know how things are working because I don't want to just give some medication based on [CDSS] saying yes or no. But what made it come to that conclusion, I need to know a bit more. So that's a clinical reasoning, isn't it? We need to find out what is the reason behind it”.* (P04 Neurology specialist nurse)

Participants queried the development of the system's intelligence, specifically how it learns over time and whether it could recognise exacerbations not identified by patients or HCPs, its potential to identify future exacerbations and if it could adapt to data from specific patient populations.

### Settings and logistics of SMaRT-PD piloting

Participants had mixed views about the optimal setting for SMaRT-PD. While regional neuroscience centres (RNC) were assumed to have better provision for care, participants noted that, given the growing prevalence of Parkinson's, the system should be adaptable to users in any setting. Smaller centres were seen to have the advantage of familiarity with patients, enabling better identification of patients suitable for a home-based pathway. However, services already operating beyond capacity, regardless of setting, were not considered suitable for introduction of a new pathway.

The (acknowledged) generalisation about rural areas and elderly patients’ ability to cope with technology persisted in discussions; participants emphasised that the home-based pathway needs to be suitable for the patient population. SMaRT-PD might be better suited to less complex patients managed at smaller centres. Implementation would need to be considerate of regional resource and cross-trust care where patients are under the management of more than one team or NHS trust.

“*The system is already relatively complex…* *with greater or lesser resource in different areas…* *it's not one Parkinson's nurse or neurology practitioner per so many people per head. It's a bit patchy. So even if the system says it triggers a review…is one area able to be much more proactive than another?”* (P07 Geriatric medicine registrar)

## Discussion

This study explored healthcare professionals’ perspectives on the potential for transitioning care pathways for people with Parkinson's from traditional clinic/hospital-based care to home-based care with remote monitoring.

Semi-structured interviews revealed that while the care pathway described by participants was relatively consistent, the settings in which it was delivered varied. Some patients were managed primarily by neurology services, while others received care through geriatric medicine. These differences in clinical oversight reflected local service configurations rather than fundamental variation in the pathway itself. Participants confirmed that the current care pathway largely aligns with NICE guidelines, with the exception of staging at diagnosis. For the most part, participants felt that the stage of Parkinson's was usually apparent, based on clinical experience and was not considered critical to determine at diagnosis stage.

Consistent with prior evidence,^40–42^ HCPs identified challenges with the existing pathway including a ‘one size fits all’ approach, delays in diagnosis, ‘patchy’ or inconsistent care, staff shortages and ensuring adequate appointment lengths and frequencies to meet patient needs. Services often spanned multiple sites or trusts, complicating care coordination and accessibility for patients. Despite these limitations, the value provided by Parkinson's services and appointments including holistic and responsive care and patient-provider relationships was emphasised. Participants felt they were providing a good service in difficult circumstances.

Most participants expressed interest in SMaRT-PD, and, in keeping with previous research into wearable technologies and self-management,^43–48^ participants identified potential benefits both to patients and to the healthcare system. Key benefits included patient empowerment, education and understanding, earlier symptom identification, reduction in non-elective admissions, correlation of symptoms with data, and clinic efficiencies.

Some remained cautious, particularly regarding the suitability of SMaRT-PD for some patient populations and concern over reduced face-to-face contact. Other research has also highlighted the potential loss of ‘doctor-patient bond’ and that wearables are not necessarily suitable for all patients.^46,49^ Participants outlined potential clinical, administrative or systemic challenges in rolling-out a home-based pathway to their patients. Views were mixed on the most appropriate setting for further implementation of the SMaRT-PD pathway. Larger specialist centres might have more resources to facilitate roll out, but smaller centres where staff knew patients well could more easily identify patients who would benefit from SMaRT-PD.

Participants wanted to see evidence of patient, carer, and staff satisfaction before they would consider implementation. Previous research into home-based care has also highlighted the importance of patient and carer satisfaction and shared decision-making.^50,51^ However, participants largely considered implementation decisions out of their hands and that the managerial or commissioner point of view would be that implementation is contingent on demonstrable cost or time savings to the NHS. Given pressures on Parkinson's services and the NHS, trying to place SMaRT-PD in an already overwhelmed service as a ‘sticking plaster’ to save time or money would be counterproductive.

Traditional barriers to implementation related to individual or interpersonal behaviours or organisational culture such as reticence to change or aversion to new technology^52–54^ were rarely cited by participants. However, an aversion to change *was* exhibited in the reluctance by some participants to consider reduction of face-to-face appointments for patients who are managing well at home. This was anticipated as a barrier not only for participants themselves but also for professional colleagues and patients. Further work might determine whether this is an attitudinal barrier to implementation or substantiated by data indicating that the removal of face-to-face appointments would leave both patients and HCPs disconnected from each other. Implementation or communication strategies should focus upon the misconception that appointments would be removed altogether and whether the ‘need’ for appointments should be based not only on clinical symptoms but on patient desire for face-to-face appointments.

This study has helped to identify where the additional generation of evidence regarding SMaRT-PD could assist implementation or further piloting, and address anticipated administrative or clinical difficulties. Future work could focus on generating cost-effectiveness data, demonstrating the pathway's impact on symptom management, and gathering patient and carer feedback; the latter increasingly important in the era of patient-centred care. Robust and generalisable evidence of patient and carer acceptance is key, not only because it was the factor most frequently cited as influencing HCPs willingness to adopt SMaRT-PD, but also because it will help identify how SMaRT-PD may need to be tailored to the needs of specific populations. Further research is also needed to determine the optimal settings for piloting (e.g., RNC or DGH) and scaling the pathway.

This study has a number of limitations. Despite purposive sampling across centre types, specialties and locations, and clear variation in service structures, our sample was largely self-selecting and therefore biased towards HCPs perhaps already comfortable and familiar with digital technology, interested in research or digital innovations and potentially more likely to be optimistic about implementation. Of thirteen directly contacted individuals, only seven participated; the remaining five were recruited via snowball sampling. Consequently, perspectives from HCPs less engaged with home-based care or research may be underrepresented. Additionally, some participants may have felt obligated to participate as initial contact came from an esteemed colleague.

We appeared to achieve consensus between participants as later interviews did not uncover new themes or data. However, the small sample limits generalisability. Efforts were made to ensure diversity of roles (neurologists, geriatricians, nurses, consultants, registrars) and geographic representation across England. We also recruited participants from North Wales due to snowball sampling. However, greater inclusion of HCPs from university hospitals, regional neuroscience centres, and devolved nations would have enhanced representativeness and may have generated new data.

Although participants readily discussed clinical implications of SMaRT-PD, insights into more systemic implementation barriers (e.g., commissioning, administrative processes) were less detailed. Further qualitative research with stakeholders involved in service implementation and commissioning, development of implementation strategies, or post-implementation evaluations would allow exploration of these issues in more depth. According to participants, patient and carer satisfaction was key to implementation. This study could therefore have been bolstered by also including the views of patients and carers to enable triangulation of the findings.

Prior knowledge of SMaRT-PD varied between participants. Despite the provision of a standardised description during interviews, some of the specifics of the SMaRT-PD system were unknown to participants (and interviewers) and will therefore have influenced some of their feedback. For example, several participants questioned whether care partners were involved in the pathway, unaware that they had contributed to its design and provided data to the CDSS. Clarification was provided where possible, though a more detailed introduction to the system may have yielded richer data.

## Conclusions

Health care professionals were generally supportive of introducing a home-based pathway for people with Parkinson's. As long as patients and carers were satisfied, HCPs thought that SMaRT-PD could provide an opportunity to concentrate limited resources where they are most needed. However, HCPs were also cautious about potentially removing face-to-face appointments completely for some patients and described the predominantly administrative barriers faced when introducing new technologies. The gap between HCP enthusiasm for a new pathway and commissioner or manager agreement to procure and implement must be bridged. To be worthwhile, the SMaRT-PD pathway would need to improve the current care pathway in a way that benefitted patients, clinicians, and the health system.

## Supplemental Material

sj-docx-1-pkn-10.1177_1877718X261440705 - Supplemental material for Transformation of Parkinson's care through SMART-PD (Self-Management, Remote monitoring and Timely review for Parkinson's Disease): Care pathway mapping with clinical experts Supplemental material, sj-docx-1-pkn-10.1177_1877718X261440705 for Transformation of Parkinson's care through SMART-PD (Self-Management, Remote monitoring and Timely review for Parkinson's Disease): Care pathway mapping with clinical experts by Nicola Howe, Chikomborero C. Mutepfa, Sara Pretorius, Jana Suklan, Abdullahi Ali, Aisling Ponzo, Phakapan Walker and Camille Carroll in Journal of Parkinson's Disease
