# Supplementary material for: Transformation of Parkinson's care through SMART-PD (Self-Management, Remote monitoring and Timely review for Parkinson's Disease): Care pathway mapping with clinical experts
Source: J Parkinsons Dis. 2026 Apr 17:1877718X261440705. Online ahead of print. doi: 10.1177/1877718X261440705 (PMC13435198; doi:10.1177/1877718X261440705)
Supplement: Supplementary file 1 [file sj-docx-1-pkn-10.1177_1877718X261440705.docx]

**SMaRT: Transformation of Parkinson’s care:**

**Interview Schedule**

Care Pathway refinement via semi-structured interviews with clinical experts

**Authors:**	Nicola Howe, Sara Pretorius, Chiko Mutepfa, Jana Suklan

**Date:**	25/03/2026

**File name:**	SMaRT_ClinicianInterviewSchedule_V2.0_2024_08_09

Newcastle HRC Introduction

Thank you for taking time to participate in this interview.

This will be a semi-structured interview *which means that we have some pre-determined questions which we will need to cover but there will also be the flexibility for more open and detailed discussion about key questions or related topics*. Before I start asking you questions, let me give you a brief overview about who we are and then take you through the verbal consent.

‘My name is … and today we are also joined by … and …. We are all methodologists working for the NIHR HealthTech Research Centre in Diagnostic and Technology Evaluation.’

‘We are independent evaluators, funded by the National Institute for Health Research, to generate evidence on diagnostic health technologies.’

‘We have expertise in the evaluation of diagnostic and digital tools used in healthcare services, our overall aim is to support understanding of how these tools can be best used in practice and help plan further research.’

‘Today we would like to discuss with you the diagnosis and treatment of Parkinson’s. Our aim is to understand current practice and healthcare resource to explore the potential role for new ways in which care can be delivered, and how they would affect decision making in this area.’

‘Thank you for reading the information sheet and returning the consent form. Can we confirm that you consent to take part, and whether you have any questions before we proceed to the interview?’

# Interview Questions

## Demographics

- What is your current job role?
  - What is your profession or speciality (nurse/neurologist care of elderly/geriatrician)
  - Where in the UK are you based (e.g. trust)?
  - What sort of hospital do you work in? (neuroscience centre, district general hospital, community specialist centre, or smaller practice)
  - How many years have you worked in …?
  - What is the size of your Parkinson’s clinic/how many patients?
  - Patient characteristics?

## Current practice

I am now going to ask you some questions about how you currently diagnose and manage people with Parkinson’s.

**What is the current care pathway for patients with suspected Parkinson’s?**

**Referrals**

- Where/to whom would an individual first go if they had Parkinson’s symptoms?
- Where/to whom would they be referred?

**Tests/decisions/confirmation of diagnosis**

- How is diagnosis confirmed- any tests- when? what order?
- Where would these tests be performed (departments/settings)?
- How is the stage of Parkinson’s determined?

**Patient management and care post diagnosis**

- Once a patient has received a Parkinson’s diagnosis, what happens next?
  - What service would manage/have oversight of their care?
- What is the average length of survival for a person diagnosed with PD?
- What is the quality of life on a scale of 1 to 10? (with 1 being the lowest and 10 being the highest rating of quality of life?)
  - Tell us about patient monitoring
  - How often would the patient be reviewed? - who decides this?
  - Who would review the patient (consultant, registrar, nurse specialist, PD practitioner)?
  - How long would each review consultation last?
  - What would each follow-up appointment involve? (tests, physical examination or questionnaires (standardised or local), conversation, change of medication etc?)
  - How involved are you in management of medication, patient usage, adherence and side effects?
  - Is everyone seen on time or is there a variability in ideal time between follow up visits?
  - What is the average wait between follow-up appointments?
  - If conversation:
    - Does this contain any motivational interviewing (client-centred counselling style for eliciting behaviour change by helping clients to explore and resolve [ambivalence](https://en.wikipedia.org/wiki/Ambivalence))? *(techniques for self-management and medication adherence)*
  - What other services would be involved in the patient’s care. On average how many services is a patient under? Which ones are the most referred to?
- How does a patient seek advice or assistance in between clinic appointments?
- Are patients with Parkinson’s ever discharged from the service?
- Does the service have any eligibility criteria e.g. degree of diagnostic certainty or age?
- **What do you think works well within the current Parkinson’s pathway?**
- **What doesn’t work well within the current Parkinson’s pathway?**

## Introduction to SMaRT-PD (Self-Management, Remote monitoring and Timely review (SMaRT-PD)

Next, we’d like to introduce you to (SMaRT-PD). This information will frame the later questions.

SMaRT-PD is clinical decision making tool (CDSS) designed to better support patient self-management through increasing knowledge and understanding of Parkinson’s, remote monitoring and the ability to trigger a healthcare contact when needed.

Prior to enrolment in SMaRT-PD the patient receives a comprehensive information and resource pack about Parkinson’s and an online, one-hour SMaRT-PD group training session.

Using a wrist worn sensor and a set of questionnaires, continuous, remote monitoring of the patient with Parkinson’s takes place for 6 days, every 6 months. The wrist-worn sensor collects data on motor symptoms (bradykinesia, dyskinesia, tremor), and immobility in relation to levodopa doses.

Questionnaires validated for use in patients with Parkinson’s assess information on motor and non-motor symptoms and quality of life for the patient and their care partner or partners.

SMaRT-PD uses these data to generate guidance for self-management for the patient to address their current symptoms, produce a report for health care practitioners and identify patients who might benefit from clinical review. Patients can also trigger contact with a healthcare practitioner at any time.

Based on the information presented:

- What are your initial thoughts about SMaRT-PD?
- Do you see any advantages to the SMaRT-PD pathway relative to the current pathway for Parkinson’s monitoring?
- Do you see any disadvantages to the SMaRT-PD pathway relative to the current pathway?
- How would the SMaRT-PD pathway change clinical decision making?
- How would SMaRT-PD fit in with your current clinical practice?
- Do you have any questions or concerns about how the wearables work?
- What could be the potential impact on average length of survival for a patient based on this intervention?
- What could be the potential impact on waiting lists or other aspects of how care is provided within your service?
- What quality of life on a scale of 1 to 10 would you expect for a patient based on this with 1 being the lowest and 10 being the highest rating of quality of life?
- Which patients would benefit most from the use of SMaRT-PD?
- Are there any patients who you feel you would not be able to use SMaRT-PD with?
- What output of SMaRT-PD do you think would be of most value in your service? *(personalised self-management guidance for patients to address their current symptoms; identification of patients who might benefit from a clinical review)*
- Why? In what way would it add value?

## Evidence Requirements

- What evidence would you want to see with SMaRT-PD in order to feel confidence in its implementation/changing the current pathway?
  - Clinical outcomes?
  - Health economics?
  - Impact on waiting lists?
  - Safety

## Barriers and Facilitators

- Are there any potential barriers to the adoption of SMaRT-PD?
- How could these be overcome?
- What factors might enable the implementation of SMaRT-PD (facilitators)?

## Further comments/questions

- Is there anything else you feel is important to tell us about Parkinson’s diagnosis that we haven’t touched upon in this interview?
- Do you have any further thoughts on SMaRT-PD?
- Do you have any feedback on questions from the interview?
